# Biomarkers of Cutaneous Leishmaniasis

**DOI:** 10.3389/fcimb.2018.00222

**Published:** 2018-06-26

**Authors:** Fariborz Bahrami, Ali M. Harandi, Sima Rafati

**Affiliations:** ^1^Department of Immunology, Pasteur Institute of Iran, Tehran, Iran; ^2^Department of Microbiology and Immunology, Institute of Biomedicine, Sahlgrenska Academy, University of Gothenburg, Gothenburg, Sweden; ^3^Department of Immunotherapy and Leishmania Vaccine Research, Pasteur Institute of Iran, Tehran, Iran

**Keywords:** cutaneous leishmaniasis, asymptomatic, *Leishmania*, biomarker, immune responses

## Abstract

Cutaneous leishmaniasis (CL) is an immune-mediated skin pathology caused mainly by *Leishmania* (*L*.) *major, Leishmania tropica, Leishmania braziliensis, L. mexicana*, and *L. amazonensis*. The burden of CL in terms of morbidity and social stigmas are concentrated on certain developing countries in Asia, Africa, and South America. People with asymptomatic CL represent a large proportion of the infected individuals in the endemic areas who exhibit no lesion and can control the infection by as yet not fully understood mechanisms. Currently, there is no approved prophylactic control measure for CL. Discovery of biomarkers of CL infection and immunity can inform the development of more precise diagnostics tools as well as curative or preventive strategies to control CL. Herein, we provide a brief overview of the state-of-the-art for the biomarkers of CL with a special emphasis on the asymptomatic CL biomarkers. Among the identified CL biomarkers so far, direct biomarkers which indicate the actual presence of the infection as well as indirect biomarkers which reflect the host's reaction to the infection, such as alterations in delayed type hypersensitivity, T-cell subpopulations and cytokines, adenosine deaminase, and antibodies against the sand fly saliva proteins are discussed in detail. The future avenues such as the use of systems analysis to identify and characterize novel CL biomarkers are also discussed.

## Introduction

### Cutaneous leishmaniasis, an ancient disease with an uneven global burden

Leishmaniases include multitudes of infectious diseases caused by the protozoa of *Leishmania* genus which pose serious public health problems in the endemic regions across 98 countries (Alvar et al., 2012). Of the two main forms of such infections, known as visceral leishmaniasis (VL) and cutaneous leishmaniasis (CL), the latter is the most common form. Leishmaniases are multifactorial diseases which their outcomes are influenced by dynamic interactions of the parasite, its reservoir(s), its vector, and eventually the immune system of its human host. The ecosystems that harbor the above factors can also affect the infectivity of the parasite. For instance, climate changes that favor expansion of deserts can provide a hospitable environment for propagation of the vectors and the reservoirs of the parasites that cause CL. The prevalence of CL in the endemic regions is on the rise due to natural favorable environmental changes, compounded by manmade influences such as global warming, deforestation, regional conflicts, mass migrations, and urbanization (González et al., 2010; Du et al., 2016). Adding to the existing complexity, recent findings have pointed to the genome instability of *Leishmania* in response to the environmental pressures by high aneuploidy turnover and haplotype selection mechanisms (Prieto Barja et al., 2017).

Earlier, it had been estimated that ~75% of global CL cases are found in 10 countries, namely Afghanistan, Algeria, Colombia, Brazil, Iran, Syria, Ethiopia, North Sudan, Costa Rica, and Peru (Alvar et al., 2012). A recent comprehensive epidemiological study based on country-level data (Table 1) has confirmed that the burden of CL falls mostly on certain developing countries in the Middle East and North Africa in the Old World as well as on a few South American and Caribbean countries in the New World. The common factors shared among all CL endemic areas are high population densities and malnutrition, combined with poor sanitary facilities (Hotez et al., 2012, 2014; Karimkhani et al., 2016). Aside from the economic burden caused by the morbidity of CL, the social stigmatization (Hurrell et al., 2016) and emotional burden due to the ulcers, especially among the inflicted women and children represent other important sequelae associated with the disease (Chahed et al., 2016; Bennis et al., 2017a,b).

**Table 1:** The burden of CL in the countries with the highest prevalence in the Old/New Worlds (Karimkhani et al., 2016).

**Burden of CL in 2013**		**Age-standardized prevalence**	**Age-standardized DALYs**
Old world	Afghanistan	8,340	87.03
	Sudan	1,925	20.24
	Syria	865	9.15
	Yemen	593	6.25
	Iraq	563	5.95
New world	Bolivia	440	4.65
	Haiti	387	4.06
	Peru	386	4.04
	Venezuela	234	2.48
	Nicaragua	155	1.52

*DALY, Disability-Adjusted Life-Years*.

### Interaction of *Leishmania* and the immune cells

*Leishmania* parasite such as *Leishmania major, Leishmania tropica, Leishmania braziliensis, L. Mexicana*, and *L. amazonensis* can be transmitted by different zoonotic and anthropozoonotic cycles that may involve domestic and wild mammalian reservoirs via a digenetic life cycle, divided into extracellular, and intracellular stages. The protozoan lives as either a promastigote form inside its female blood-feeding sand fly of *Diptera* order, belonging to *Phlebotomus* (*Ph*.; in the Old World) or *Lutzomyia* (*Lu*.; in the New World) genera or an amastigote form inside parasitophorous vacuoles within the phagocytic cells (mostly macrophages) of its vertebrate host (Lestinova et al., 2017). The infection turns into ulcerated skin lesions when the cell-mediated immune responses fail to eliminate or control the resident parasites inside the phagocytic cells.

Phagocytic cells such as neutrophils are deemed to play a crucial role during the infection which can lead to either survival or destruction of the immunologically-evasive *Leishmania* parasites. Soon after the parasite entry, neutrophils are massively recruited to the site of infection. These cells mediate their defense functions through two different approaches: (i) phagocytosis and killing of the parasites (ii) formation of neutrophil extracellular traps (NETs) and releasing different microbicidal agents associated with DNA backbone (Brinkmann et al., 2004). Through NETs, neutrophils are able to entrap and inhibit the spread of the parasites (Zawrotniak and Rapala-Kozik, 2013). Based on *in vitro* studies, it has been shown that *L. major* and *Leishmania donovani* may survive inside the neutrophils by inhibiting the process of granule fusion with the parasites contained inside the phagosomes (Gueirard et al., 2008; Hurrell et al., 2016). Furthermore, parasites such as *L. major* and *L. braziilensis* have been shown to be capable of resisting the microbicide activities associated with NET formation through endonuclease digestion, derived either from the parasites or the sand fly components. Therefore, through different approaches, the parasite succeeds to survive inside the neutrophils (Regli et al., 2017). Depending on the *Leishmania* species, neutrophils can influence the disease outcome and act as important modulators of leishmaniases (Hurrell et al., 2016). Moreover, skin resident macrophages and dendritic cells (DCs) are shown to be capable of presenting the processed antigens of the amastigotes to T cells; albeit each using different machineries (Cecílio et al., 2014).

Interestingly, after encounter with *Leishmania* parasites, the biological behaviors of the phagocytes are altered so much as the macrophages become the host cell for the parasite and DCs appear to become mainly responsible for T cell priming and induction of protective immunity (von Stebut and Tenzer, 2017). There is no clear evidence on the fate of *Leishmania* parasites within the DCs. The importance of such antigen-presenting cells in parasite maintenance and induction of long term memory is also evident for the healed patients of CL and may also have functions for the asymptomatic population. A more comprehensive understanding of the versatile functions of DCs in CL and their interplay with other immune cells may provide new insights into underlying immune mechanisms that control CL.

## Biomarkers of CL

In order to evaluate and compare curative or preventive strategies on people affected by CL, the researchers require defining biomarkers as indicators of normal biological processes, as well as tools to detect them. The application of biomarkers with respect to diseases such as CL can be categorized into different approaches such as diagnostics, prognosis and monitoring the disease progression or the outcome of clinical interventions (Mayeux, 2004). The methodology being used to assess the biomarkers should ideally be safe and sensitive, capable of producing consistent results for different genders and ethnic groups, objectively (Theppeang et al., 2008; Strimbu and Tavel, 2010). Since CL is mostly prevalent in the developing countries, a tool to evaluate a CL biomarker should also be relatively affordable for the afflicted societies.

Biomarkers are often divided into direct and indirect categories in the literature. Concerning CL, factors, products or conditions resulted directly from the infection can be considered as a direct biomarker. Whereas, host's quantitative or qualitative biological reactions due to the leishmanial infection can be considered as indirect biomarkers. Identifying biomarkers associated with diagnostic, treatment, and especially disease outcome may help to develop new ideas or tools for better understanding of the mechanisms behind the protective immune responses in CL. A comprehensive systematic review of potential pharmacodynamic biomarkers of different forms of leishmaniases has been published in recent years (Kip et al., 2015). While few studies on biomarkers associated with VL caused by *Leishmania infantum* and *L. donovani* have been conducted (Ibarra-Meneses et al., 2016, 2017), little is known about CL biomarkers in general and asymptomatic CL biomarkers, in particular. Although not exhaustive, we herein will briefly discuss the state-of-the-art for markers of CL. The putative biomarkers based on CL studies, divided into direct and indirect markers, are summarized in Table 2.

**Table 2:** Putative biomarkers of CL.

**Direct Markers**	**Infection Detection**				
	Parasite burden evaluation				
	Parasite detection				
**Indirect Markers**	**Indicators of delayed-type hypersensitivity**	**Enzymes activities**	**Cytokines measurements**	**Activities of T-Cells populations**	**Antibodies against sand fly saliva proteins**
	LST	ADA	IL-10 mRNA (lesion)	Th1	anti-SGH
		Arginase	TNF-α (plasma)	Th2	anti-PpSP32
			TNF-α mRNA (lesion)	T_reg_	anti-LJM11
			IL-6 (lesion)	Th17	anti-LJM17
			IL-12 (lesion)		
			IL-17 (lesion)		

*LST, Leishmanin Skin Test; ADA, Adenosine Deaminase; T_reg_, Regulatory T Cells; SGH, Salivary Gland Homogenate*.

### Detection of infection

The determination of presence of a CL-causing *Leishmania* parasite in tissues can be considered as the most direct biomarker of CL. However, diagnosis of CL is not an easy task due to lesion variation in term of severity, clinical appearance, and duration; therefore, development of sensitive diagnostic biomarkers is in high demand (Akhoundi et al., 2017). Occasionally, diagnostic decision is also complicated due to the similarity of the clinical symptoms, in cases such as bacterial ulcers, leprosy, sarcoidosis, and lupus vulgaris. Traditionally, definitive diagnosis was based on visualization of *Leishmania* on a direct smear, followed by culturing and animal inoculation. Later, molecular diagnostic methods gained prominence due to their rapidity, sensitivity, and specificity which enabled the investigators to discriminate even among different *Leishmania* species by using different species-specific probes. There are also non-invasive sampling methods in which the parasite DNA is isolated by sequential tape strips, followed by parasite detection, using PCR (Taslimi et al., 2017). Although there is no specific gold standard technique for detection and diagnosis of *Leishmania* infection, the complete sequencing of several *Leishmania* genomes can open new windows for revealing precise diagnostic biomarkers in future (Van der Auwera et al., 2014). A few indirect biomarkers of CL which have been clarified so far are mentioned below.

### Delayed-type hypersensitivity

The parasite propagation inside macrophages usually leads to typical ulcers which may last for a year. After healing, such ulcers leave disfiguring scars on the skin. Due to the importance of cell-mediated immunity (CMI) in the outcome of CL (Sacks and Noben-Trauth, 2002), the state of CMI for the suspected CL patients is generally evaluated by testing delayed-type IV hypersensitivity [DTH; (Turk, 1979)] reaction against the leishmanial antigens. Such tests can be performed by Leishmanin skin test (LST; previously known as Montenegro test) that indicates prior encounter of the host immune system with the parasite. A positive DTH response is evidenced by a small induration which forms 72 h after a 100-μl intradermal injection of Leishmanin reagent (a phenolized cultured *L. major* promastigotes). LST remains a powerful biomarker to distinguish the subpopulations with respect to asymptomatic CL. Moreover, LST results can be interpreted as an indicative of the developed immunological memory (Andrade-Narvaez et al., 2016).

### T-cell subpopulations and the importance of cytokines as putative biomarkers

Since 1980s, investigators have been trying to figure-out distinct functions of thymus-derived T cells and their cytokine profiles with respect to leishmaniases. These murine-based studies, performed with mostly *L. major* infections, led to the proposition of two counter-regulatory CD4+ T-cell subpopulations, known as Th1 and Th2 which are accounted for controlling resistance and susceptibility to the infection, respectively (Alexander and Brombacher, 2012). However, no such clear-cut Th1/Th2 phenotype has so far been demonstrated in human CL studies. Exacerbated Th1 cell-mediated immune responses during CL, such as excessive secretion of pro-inflammatory IFN-γ cytokine, have been shown to cause tissue damage and are assumed to contribute to the lesion progress (Maspi et al., 2016).

Besides the importance of Th1 and Th2 functions with respect to immunity to CL, two other T cell subpopulations, namely regulatory T cells (T_reg_) and Th17 cells have also been identified to regulate immunity to CL. T_reg_ are known to function as the sustainers of tolerance and preventers of excessive damages during the inflammatory responses (Suffia et al., 2005). Evidence indicates that in CL caused by *L. braziliensis* and *L. guyanensis*, functional T_reg_ are recruited to the lesion sites (Campanelli et al., 2006; Bourreau et al., 2009). Th17 cells, on the other hand, have been proposed to orchestrate a balance between the pro- and the anti-inflammatory cytokines during CL and also to recruit neutrophils to the site of infection (Gonçalves-de-Albuquerque et al., 2017).

Among the cytokines explored in CL studies, IL-10 appears to serve as a putative biomarker which can exhibit the treatment failure. It has been shown that mRNA level of this regulatory cytokine in CL lesion is positively correlated to unresponsiveness to the treatment (Louzir et al., 1998; Bourreau et al., 2001). The mRNA level of pro-inflammatory cytokine TNF-α in CL lesion biopsy has been shown to be positively correlated with the lesion size (Louzir et al., 1998). While the elevated serum levels of TNF-α has been suggested to be associated with the severity of mucocutaneous leishmaniasis, whether or not this holds true for CL has been a matter of debate (Barral-Netto et al., 1991; Castes et al., 1993; Vouldoukis et al., 1994; Da-Cruz et al., 1996; Kocyigit et al., 2002a,b). Furthermore, IL-6 transcript in CL lesion biopsies and IL-6 protein in the patients' sera have been associated with the lesion size in CL patients (Louzir et al., 1998; Kocyigit et al., 2002a). Unhealed lesions as well as the lesion duration were shown to be correlated with IL-12 p40 (Melby et al., 1996; Louzir et al., 1998).

### Enzymes

Adenosine deaminase (ADA) found in macrophages mediates deamination of the anti-inflammatory nucleoside adenosine to inosine. The level of ADA has been shown to be significantly increased in sera and lymphocytes of CL patients (Erel et al., 1998). In a recent study, it has been shown that serum ADA activity in patients with active VL and post kala-azar dermal leishmaniasis (PKDL) were significantly higher than their respective treated cases as well as the healthy individuals. The ADA activity in PKDL has been found to be decreased gradually during the different phases of treatment which suggests that this parameter can act as a marker of pathogenesis and prognosis of the disease (Vijayamahantesh et al., 2016).

L-arginine is a conditionally essential amino acid that plays a role in many metabolic pathways and serves as a common substrate for both arginase and nitric oxide synthase (NOS). In CL, arginine acts as a double-edged sword so that while it is needed for nitric oxide (NO)-mediated parasite killing, it can promote polyamine-mediated parasite replication (Gogoi et al., 2016). The induction of arginase activity in CL lesions causes the depletion of arginine and hence the reduction of NO content. Altogether, arginase limits the availability of arginine and becomes an agent of immune suppression and impairment of T-cell responses. In line with this notion, it has been shown that the level of arginase activity is significantly increased in sera of CL patients (França-Costa et al., 2015). Moreover, in patients infected with either *L. major* or *L. tropica*, the levels of arginase activity in the lesion of those who had acute CL (duration ≤ 1 year) were higher than those with chronic CL (duration ≥2 years) and also in the skin of the uninfected people (Mortazavi et al., 2016). Interestingly, arginase activity in *L. major* promastigotes, isolated from CL patients has been shown to be significantly higher than the activity of this enzyme in the non-pathogenic strain of this parasite (Badirzadeh et al., 2017).

### Antibodies raised against sand fly saliva

Female phlebotomine sand flies are the only natural vectors of *Leishmania* species. The importance of sand fly saliva for the establishment of the infection on the feeding sites is well-documented (Maroli et al., 2013). The skin damage caused by the biting mechanics unleashes a hostile environment for the blood-feeding insect through hemostasis, inflammation and immune responses. The parasite in such unfavorable milieu is deemed to be counteracted by anti-hemostatic, anti-inflammatory and immunomodulatory components of the sand fly saliva. Several immunomodulatory components common among different sand fly saliva, and in particular enzymes such as apyrase, hyaluronidases, and endonucleases have been shown to regulate both the innate and the adaptive immune responses (Lestinova et al., 2017).

It has been shown that experimental exposure of mice, dogs and humans to sand fly bites induces antibody production after a few weeks; although in such cases the magnitude of the response is dependent on the number of sand fly bites (Abdeladhim et al., 2014). The immune response to specific sand fly saliva component could act as reliable biomarkers of vector exposure. Therefore, identification and characterization of salivary antigens which are specific to a particular vector species can be utilized as an exposure biomarker. The salivary antigen PpSP32 is the immunodominat target of antibody response to *Phlebotomus papatasi* bites in human. The antibody against recombinant PpSP32 (rPpSP32) has been recently tested as a putative biomarker of exposure to sand fly in Tunisia (Marzouki et al., 2012). However, the anti PpSP32 response has not been documented in dogs immunized with saliva of *Ph. perniciosus* (Marzouki et al., 2015). There are other studies in South America where it has been shown that antibody against LJM11 and LJM17 from *Lutzomyia longipalpis* saliva could act as putative biomarkers of vector exposure for both humans and dogs (Teixeira et al., 2010). These two proteins belong to the yellow family and are abundant in sand fly and absent from saliva of mosquitoes (Xu et al., 2011; Abdeladhim et al., 2014). It is also noteworthy that the kinetics and duration of antibody responses could also be considered as a putative biomarker of exposure to the vector or the parasite.

### Putative biomarkers for asymptomatic CL infection

Although the minority of people living in the endemic areas succumb to CL and manifest ulcers, a significant proportion of individuals exhibit a positive DTH response without any skin ulcer. The underlying mechanism through which asymptomatic individuals control the infection is not fully understood. Understanding the immunological basis of CL asymptomaticity represents an unmet need, which if negotiated, could inform the development of tools to detect and control CL. Notwithstanding, it is becoming increasingly clear that addressing multifactorial complex diseases such as leishmaniases which are affected by diverse immunological, genetic, and environmental factors would benefit from an unbiased systems analysis approach.

It is well-documented that after clinical cure of certain intracellular pathogens, such as herpes viruses, *Mycobacteria, Chlamydia*, and *Trypanosoma*, a long-term persistence of these pathogens can be detected in the body (Mendonça et al., 2004). Due to the lack of adequate diagnostics tools, it is however challenging to differentiate people with persistent *Leishmania* infection from those with immunologically-sensitizing exposures (Andrade-Narvaez et al., 2016). The existence of live *Leishmania* parasites of various strains has been reported after clinical cure by chemotherapy. In one study, by applying precise molecular approaches, the presence of live parasites has been confirmed in mucocutaneous leishmaniasis caused by *Leishmania* species of the *Viannia* subgenus. The presence of *Leishmania* has been demonstrated in blood monocytes, tonsils and normal skin of clinically-cured as well as the asymptomatic individuals using amplification of 7SLRNA gene. As mentioned earlier, the immune responses are playing outstanding roles in this regard. For instance, the monocyte-derived macrophages from people with asymptomatic CL due to *L. braziliensis* are shown to control the parasite much stronger than macrophages from patients with lesions. These data besides many others may show the intrinsic differences between the innate immune responses (Giudice et al., 2012; Scorza et al., 2017).

The frequency and suppressive capacity of regulatory T cells (CD4^+^CD25^high^ FoxP3+) have been shown to be comparable in the peripheral blood of the healed and the asymptomatic individuals in an endemic area of *L. major* infection in Iran. However, mRNA level of the T_reg_ transcription factor *FOXP3* was shown to be higher in the asymptomatic group compared to the symptomatic patients. The mRNA level of IL-17 transcription factor *RORC*, on the other hand, was not significantly different between the groups (Bahrami et al., 2014).

Recently, a cross-sectional immune profiling study on PBMC of individuals living in endemic areas of *L. major* transmission in Tunisia with documented exposure history was conducted (Kammoun-Rebai et al., 2016). The participants were subdivided according to their LST responses and the presence of scar, into three groups of healed, asymptomatic and naïve individuals. Significant *Leishmania*-specific responses in LST+SCAR+ and LST+SCAR- individuals, but not in LST-SCAR- (naïve subjects), were substantiated. Similarly, the levels of IL-2, IL-12p70, IL-13, and IL-18 were shown to be significantly higher in all LST+ individuals but not in LST- cohort. Once validated, such biomarkers could be potentially used to stratify patients for different clinical/sub-clinical manifestations of the disease.

Although LST is considered safe, its application requires an invasive injection of the killed parasite in a phenol solution. As a potential alternative to LST, salivary gland homogenates (SGH), or proteins thereof may be used to determine the risk of contact, vector surveillance and as such could inform the disease management programs (Oliveira et al., 2013a). In humans, the bite of sand flies has been shown to induce DTH at the biting sites and can generate specific antibody responses, detectable in serum (Oliveira et al., 2013b). As such, DTH and specific antibodies can presumably be also used as markers of exposure to the vector's bite in humans and animals. As mentioned above, the recombinant PpSP32 from *Ph. papatasi* could act as a serum biomarker of exposure (Marzouki et al., 2012). Hence, identification of different biomarkers from various infective sand flies combined with the development of simple and robust tools such as those based on finger-prick capillary sampling methods, and autoreactive strips with already-fixed rPpSP32, could facilitate the screening of the target population in the endemic areas.

### Asymptomatic CL patients serve as potential reservoirs

Characterization of *Leishmania* in asymptomatic infection is technically challenging due to the limitation in the detection of low number of the parasites. Importantly, the low number of the parasites present in the skin of the asymptomatic individuals may act as an infection reservoir. PKDL is a good example of such situations in which an asymptomatic infection can convert into PKDL (Saha et al., 2017). Therefore, development and use of precise and sensitive molecular tools could help identifying asymptomatic individuals with low-level of infection, which could in turn facilitate the development of measures to control CL.

There is a dearth of documents regarding the parasite detection in either skin or blood of the asymptomatic patients of the Old World CL. Of note, the detection of *L. tropica* and *L. major* in healed CL individuals with various durations after the recovery has recently been reported (Taslimi et al., 2017). The presence of *Leishmania* parasites in unaffected skin and peripheral-blood monocytes of American CL due to *Leishmania* (*Viannia)* species has also been reported (Vergel et al., 2006). The *Leishmania* kinetoplast DNA has been detected by PCR from scar and peripheral blood, several years after the healing. In addition, the presence of *Leishmania* (*Viannia*) parasites in tissues and its availability in tissues accessible to sand flies were demonstrated in that study. By considering two body sites consisting of the border of an active lesion and a normal skin site, viable parasites were detected in the unaffected skin of these individuals (Rosales-Chilama et al., 2015). Considering the aforementioned evidence, and the similarities of the immune responses between the healed and the asymptomatic populations in CL endemic areas, it is plausible that *Leishmania* species persist inside the skin of the asymptomatic individuals. To substantiate this, efforts should be made to analyze the skin and blood of the asymptomatic subjects with confirmed DTH, in the highly endemic areas.

## Future directions

While few indicative biomarkers of CL infection and immunity have been proposed using conventional approaches, systems-level data integrating different layers of information on host response to CL infection are scarce. Such information can provide a comprehensive understanding of the disease and may presumably identify key biomarkers of CL disease and immunity. The recent technological advances in high-throughput omics technology combined with systems biology approaches have begun to provide new insights into pathogenesis of different diseases, and to unravel the molecular signatures of vaccine-induced responses in humans. Recently, whole genome transcriptomics analysis has been applied to CL. We have reported the transcript changes in the lesion of patients infected with *L. tropica* as compared with the healthy normal skin (Masoudzadeh et al., 2017). We have identified key immunobiological pathways that are regulated following *L. tropica* infection during the acute phase of the disease. Another recent transcriptomics study of lesion biopsies from *L. braziliensis* patients has identified B cell activation and immunoglobulin transcript signatures in the lesions, depending on the presence or absence of the parasite transcripts (Christensen et al., 2016). Based on the rapid pace of development in the field, it is envisaged that more putative biomarkers with the power to predict the outcome of CL infection caused by different *Leishmania* species will be identified (Patino and Ramírez, 2017). In interpreting the biomarker data, caution should however be exercised as the identified biomarkers may merely serve as surrogate markers of infection or immunity, and as such may not necessarily play a causal role in pathogenesis of or immunity to CL.

Once available, such key information on biomarkers could inform the design of rational diagnostics and intervention strategies to control CL. In particular, this can help to screen the populations living in the endemic areas for asymptomatic individuals, which can in turn provide invaluable information on how gender, ethnic or geographical factors would affect the asymptomaticity. To achieve this, accurate, non-invasive and rapid methods with the ability to distinguish latent CL from the asymptomatic CL are needed. It should be noted that more awareness at global and national levels is also required to pave the way for developing cost-effective diagnostics and intervention strategies for the expanding inflicted populations.

## Author contributions

FB, AH, and SR drafted the manuscript. All the authors provided critical feedback on the manuscript prior to publication and have agreed to the final content.

### Conflict of interest statement

The authors declare that the research was conducted in the absence of any commercial or financial relationships that could be construed as a potential conflict of interest.
